# Detecting Parkinson’s disease from sustained phonation and speech signals

**DOI:** 10.1371/journal.pone.0185613

**Published:** 2017-10-05

**Authors:** Evaldas Vaiciukynas, Antanas Verikas, Adas Gelzinis, Marija Bacauskiene

**Affiliations:** 1 Department of Electrical Power Systems, Kaunas University of Technology, Kaunas, Lithuania; 2 Department of Information Systems, Kaunas University of Technology, Kaunas, Lithuania; 3 Center for Applied Intelligent Systems Research (CAISR), Halmstad University, Halmstad, Sweden; Universita degli Studi di Pisa, ITALY

## Abstract

This study investigates signals from sustained phonation and text-dependent speech modalities for Parkinson’s disease screening. Phonation corresponds to the vowel /*a*/ voicing task and speech to the pronunciation of a short sentence in Lithuanian language. Signals were recorded through two channels simultaneously, namely, acoustic cardioid (AC) and smart phone (SP) microphones. Additional modalities were obtained by splitting speech recording into voiced and unvoiced parts. Information in each modality is summarized by 18 well-known audio feature sets. Random forest (RF) is used as a machine learning algorithm, both for individual feature sets and for decision-level fusion. Detection performance is measured by the out-of-bag equal error rate (EER) and the cost of log-likelihood-ratio. Essentia audio feature set was the best using the AC speech modality and YAAFE audio feature set was the best using the SP unvoiced modality, achieving EER of 20.30% and 25.57%, respectively. Fusion of all feature sets and modalities resulted in EER of 19.27% for the AC and 23.00% for the SP channel. Non-linear projection of a RF-based proximity matrix into the 2D space enriched medical decision support by visualization.

## Introduction

Parkison’s disease (PD) is the second most common neurodegenerative disease after Alzheimer’s [1] and it is anticipated that the prevalence of PD is going to increase due to population ageing. The loss of dopaminergic neurons can reach up to 50% at the time of clinical diagnosis [2] and rapidly increases completing by 4 years post-diagnosis [3]. Any neuroprotective strategies that may emerge in the near future could be too late to effectively slow down the neurodegenerative process. Therefore, early objective diagnostic markers are critically needed. Amongst many other symptoms, PD manifests itself through speech disorders, which can be observed as early as 5 years before the diagnosis [4]. Investigations show that Parkinsonian vocal dysfunction can be characterized by: reduced vocal tract volume and reduced tongue flexibility, significantly narrower pitch range, longer pauses and smaller variations in pitch range, voice intensity level, and articulation rate. Therefore, automated acoustic analysis is considered by many researchers as an important non-invasive tool for PD screening. To this end, acoustic analysis aims at solving either regression or classification task: PD severity evaluation based on vocal function assessment from audio samples [5–8], as in the Interspeech 2015 computational paralinguistics challenge, or early detection of PD by learning to classify audio samples into healthy control (HC) or PD cases [9–17].

Recent computational and electronic advancements have made it possible for researchers to explore ambitious concepts such as smart homes or personalized medicine, and to bring us closer to the realization of ambient intelligence in our daily environments [18]. Ambient intelligence has potential to provide low-cost healthcare monitoring in an unobtrusive way and enhance healthcare domain dramatically. Usage of hand-held device, such as smart-phone, for non-invasive measurements is getting increased attention from the researchers. Prominent examples of this direction with respect to PD is Johns Hopkins [19] and the mPower [20] studies. In these studies performance of PD detection using smart-phone internal microphone is not compared to the detection using professional microphone. Therefore, it remains unclear how much the quality of the recording channel influences performance.

Size of previously used databases is a major problem undermining reported estimates of PD detection performance. Very small datasets (usually less than 60 PD cases) are used in most studies performed so far with various success: 98.6% detection accuracy was obtained by [11] using /*a*/ phonation from 33 PD and 10 HC subjects, 92% detection accuracy was achieved by [12] using /*e*/ phonation from 20 PD and 20 HC subjects, 71.6% detection accuracy was reported by [13] using /*i*/ phonation from 50 PD and 50 HC subjects. Experiments of [21] using /*a*/ phonation from 50 PD and 50 HC subjects achieve 82% and 90% accuracy for males and females, respectively. Vasquez-Correa et al. [15] used running Spanish speech recorded in non-controlled noise conditions from a set of 14 PD and 14 HC subjects to detect PD. Voiced and unvoiced segments of the signals were analysed separately and different sets of audio features were considered, achieving 86% and 99% detection accuracy for voiced and unvoiced frames, respectively. Expanded corpus, containing 170 German speakers (85 PD and 85 HC), 100 Spanish speakers (50 PD and 50 HC), and 35 Czech speakers (20 PD and 15 HC), having recordings of texts and monologues, was used by [16] and the energy content in the transitions between voiced and unvoiced segments was estimated. Using read texts the detection accuracy ranged from 91% to 98%, depending on the language, whereas using monologues accuracy exceeded 98% for all the three languages. Their seminal research [17], based on probably the largest number of PD subjects (88 German speakers in the trilingual corpora), recommends splitting of speech recording into voiced / unvoiced parts and reports accuracies ranging up to 99%.

Another common problem in some of the previous studies [8, 9, 12–14, 22–25] is the lack of declaration that leave-one-subject-out [17], also known as leave-one-individual-out [10], validation scheme was respected. The importance of disjointedness with respect to subjects arises when a subject is represented by several recordings and all subject’s recordings should be included either in a training or in a testing sample. For example, conformity to leave-one-subject-out validation scheme in [12, 13] could have been lacking, since methodological guidelines of [26] they follow do not stress the importance of disjointedness on the subject-basis. Meanwhile, the new publication of similar authors [21] do not refer to guidelines of [26] anymore, but explicitly declare that “each subject is in a different test fold, and the same subject never is in both test and train groups”. The Oxford PD detection dataset, donated by [9] and available in the UCI data repository, contains 22 pre-calculated features (signal amplitude and fundamental frequency perturbation measures, signal-to-noise ratios) for a set of 24 PD and 8 HC subjects, each having ∼6 recordings with /*a*/ phonation. This dataset was used by many researchers, resulting in detection accuracies ranging from 91.4% [9] or 91.8% [22] up to 99.49% [14] and even reaching 100% [23–25]. High PD recognition accuracy from voice recordings, reported in these researches, could be suspected to be achieved due to the lack of conformity to leave-one-subject-out scheme. Accuracy of 79.17% was obtained by [8] when categorizing the Oxford dataset into the healthy class and three classes of PD of different severity, but it remains not clear, if disjointedness with respect to subjects was followed. Comparison of validation approaches in [10] using the Oxford dataset reveal 81.53±2.17% accuracy for leave-one-individual-out versus 92.75±1.21% accuracy for leave-one-out sampling. Naranjo et al. [27] suggested using a subject-based Bayesian approach to deal with dependency in a “replicated measure-based design” (several recordings from one subject), demonstrating 75.2% accuracy on a dataset of 40 PD and 40 HC subjects.

The main emphasis of the related work remains on the extraction of various feature sets. Some researchers use large sets of audio features with an aim to comprehensively characterize recordings [15], including the renowned cepstral coefficients such as Mel-frequency (MFCCs) or perceptual linear predictive (PLPCCs), while others adopt only “clinically useful” measures or apply feature selection [11] to arrive at a compact set of audio descriptors. Comprehensive review of the related work was recently compiled by [17]. There is a lack of studies comparing performance of popular audio feature sets on the same dataset and considering fusion of feature sets from several modalities. Due to variety of datasets and performance assessment procedures used in different studies, and also due to different preferences and approaches for feature engineering, the question concerning the discriminatory power of various well-known audio feature sets remains unanswered. We try to address the aforementioned problems by exploring 18 diverse collections of audio descriptors on the same database recorded through two channels—acoustic cardioid (AC) and smart phone (SP) microphones. Unimodal and multimodal decision-level fusions of individual feature sets from phonation, speech, and voiced / unvoiced modalities are considered for a robust and accurate PD detection. Variable importance as a mean decrease in detector’s accuracy is reported. Finally, a convenient solution regarding data visualization for medical decision support is demonstrated.

## Phonation and speech data

Two vocal tasks were recorded in a sound-proof booth and treated as separate modalities—phonation and speech. Phonation modality contains a sustained voicing of vowel /*a*/ vocalized at a comfortable pitch and loudness level for at least 5 s and repeated 3 times. Speech modality contains a single pronunciation of a phonetically balanced sentence in a native Lithuanian language,—“turėjo senelė žilą oželį”,—which translates into “granny had a little greyish goat”. Speech recording was split using Praat software into voiced / unvoiced parts, which were treated as additional modalities in experiments. Audio samples were recorded using two channels simultaneously—acoustic cardioid (AKG Perception 220, frequency range 20–20000 Hz) and a smart phone (an internal microphone of Samsung Galaxy Note 3). Both microphones were located at ∼10 cm distance from the mouth. The audio format was mono PCM wav (16 bits at 44.1 kHz sampling rate). A mixed gender database was collected where 99 subjects had both AC and SP recordings. One PD male subject had AC speech recording missing, therefore, fusion of modalities for AC channel was possible only for 98 subjects. Full details are in Table 1.

**Table 1:** Summary of the database: Numbers correspond to the count of subjects (recordings).

	Phonation		Speech		Fusion	
	AC	SP	AC	SP	AC	SP
HC male	11 (33)	11 (33)	11	11	11 (33)	11 (33)
HC female	24 (72)	24 (72)	24	24	24 (72)	24 (72)
HC total	35 (105)	35 (105)	35	35	35 (105)	35 (105)
PD male	30 (89)	30 (90)	29	30	29 (85)	30 (90)
PD female	34 (101)	34 (102)	34	34	34 (101)	34 (102)
PD total	64 (190)	64 (192)	63	64	63 (186)	64 (192)
**Total**	99 (295)	99 (297)	98	99	98 (291)	99 (297)

Notes. Subject: PD—Parkinson’s disease patient, HC—healthy control subject. Microphone: AC—acustic cardioid, SP—smart phone.

## Audio feature sets

Information from an audio recording of phonation or speech signal can be extracted using a variety of signal analysis techniques. Computed measures are commonly known as features. Full list of audio feature sets used in this study is provided in Table 2. All the feature sets were published before and most have publicly downloadable feature extractors. With regard to the amount of signal used for calculations, features can be categorized into:

- global, long-term or high-level descriptors;
- local, short-term or low-level descriptors (LLDs).

**Table 2:** List of the individual feature sets.

#	Feature set name	Size	Reference
1	avec2011	1941	[28]
2	avec2013	2268	[28]
3	emo_large	6552	[28]
4	emobase	988	[28]
5	emobase2010	1582	[28]
6	IS09_emotion	384	[28]
7	IS10_paraling	1582	[28]
8	IS10_paraling_compat	1582	[28]
9	IS11_speaker_state	4368	[28]
10	IS12_speaker_trait	5757	[28]
11	IS12_speaker_trait_compat	6125	[28]
12	IS13_ComParE	6373	[28]
13	Essentia descriptors	1915	[29]
14	MPEG7 descriptors	527	[30]
15	KTU features	1267	[31, 32]
16	jAudio features	1794	[33]
17	YAAFE features	1885	[34]
18	Tsanas features	339	[35]

The local features are obtained by dividing a recording into short overlapping frames and applying an algorithm that computes a respective LLD for each frame. LLDs subsequently can be compressed into high-level descriptors by computing various statistical functionals. The feature sets # 1–12 had their own predefined choice from 42 statistical functionals. Statistical functionals for the feature sets # 13–17 correspond to the following 13 characteristics: minimum, maximum, mean, median, lower quartile (*Q*_*lo*_), upper quartile (*Q*_*up*_), trimean ($\frac{2\cdot median+Q_{lo}+Q_{up}}{4}$), standard deviation, inter-quartile range, lower range (*median* − *Q*_*lo*_), upper range (*Q*_*up*_ − *median*), skewness, and kurtosis. The feature set # 18 uses mostly mean and standard deviation.

### OpenSMILE features

The feature sets # 1–12 are computed using preconfigured setups available in the openSMILE [28] toolkit (version 2.2 RC 1). Name of each feature set is identical to the name of the configuration (.conf) file. Most of these setups are quite similar, therefore, for illustration, only contents of emobase.conf are specified in Table 3. The feature set emobase, introduced for emotion recognition, contains 26 LLDs and also the 1st derivative (delta or velocity) of each LLD. To summarize various aspects of frame-based data distribution for each LLD and its delta, a collection of statistical functionals is applied. The overall size of the feature set is 988 features = (26 LLDs + 26 deltas) × 19 functionals.

**Table 3:** Overview of the emobase.conf file settings.

Low-level descriptors	Statistical functionals
intensity, loudness, pitch, pitch envelope, 12 MFCCs, 8 frequencies of line spectral pairs, probability of voicing, zero-crossing rate	min (or max) value and its relative position in a signal, range, arithmetic mean, standard deviation, skewness, kurtosis, 3 quartiles, 3 inter-quartile ranges, 2 linear regression coefficients, linear and quadratic error

The file emobase.conf contains these processing-related settings:

- pitch and pitch envelope are estimated using pre-emphasis (of 0.97) and overlapping (by a step of 10 ms) Hamming windows (of 40 ms duration);
- other LLDs are obtained without pre-emphasis and the signal is windowed into overlapping (by a step of 10 ms) Hamming windows (of 25 ms duration).

Computed LLDs are smoothed with a simple moving average filter (window size = 3) before compressing by statistical functionals.

### Essentia descriptors

The feature set # 13 was computed using an open-source C++ library for audio analysis—Essentia [29] (version 2.1 beta 2)—and its out-of-the-box feature extractor streaming_extractor_freesound.exe (version 0.3). The lowlevel and sfx descriptor types were used and the tonal and rythm descriptor types were discarded (due to the fact that analysed signals are human voice and speech but not music). A detailed list of 1915 (17 global + 146×13 local) descriptors:

- 1 global descriptor of the lowlevel type—average loudness;
- 16 global descriptors of the sfx type—5 temporal (centroid, decrease, kurtosis, skewness, spread), 4 morphological (the ratio between the index of the maximum value of the envelope of a signal and the total length of the envelope, the ratio of the temporal centroid to the total length of a signal envelope, the weighted average of the derivative after the maximum amplitude, the maximum derivative before the maximum amplitude), pitch centroid, strong decay, flatness, log attack time of a signal envelope, the ratio between the index of the maximum value of the pitch envelope of a signal and the total length of the pitch envelope, the ratio between the index of the minimum value of the pitch envelope of a signal and the total length of the pitch envelope, and the ratio between the pitch energy after the pitch maximum to the pitch energy before the pitch maximum;
- 141 local descriptors of the lowlevel type—spectral energy in 77 bands (28 frequency bands, 4 bands of low/mid-low/mid-high/high frequencies, 18 ERB bands, 27 Bark bands), 3 statistics of spectral energy in Bark bands (kurtosis, skewness, spread), 13 MFCC, 13 GFCC (using Gammatone filterbank), 15 spectral (energy, entropy, complexity, centroid, strong peak, crest, Masri-Bateman high frequency content measure, RMS, roll-off, decrease, flatness in dB, flux, kurtosis, skewness, spread), 6 spectral contrasts, 6 spectral contrast valleys, 3 pitch-related (pitch, instantaneous confidence of pitch, salience of pitch), 3 silence rates (20 dB, 30 dB, 60 dB), dissonance, and zero-crossing rate;
- 5 local descriptors of sfx type—3 tristimulus values, inharmonicity, and odd-to-even harmonic energy ratio.

### MPEG7 descriptors

The feature set # 14 was composed from MPEG-7 standard-based descriptors which were extracted using the Java library MPEG7AudioEnc [30] (version 0.4 RC 3). The MPEG-7 audio standard defines normative for audio content description as a comprehensive form of meta-data, enhancing searchability of multimedia content. A detailed list of 527 (7 global + 40×13 local) descriptors:

- 7 global descriptors—4 harmonic spectral (centroid, deviation, variation, spread), 2 centroid (spectral, temporal), and log attack time;
- 40 local descriptors—36 audio spectrum (24 flatness, 10 envelope, centroid, spread), 2 audio harmonicity, audio fundamental frequency, and audio power.

### KTU features

The feature set # 15 was introduced for voice pathology screening by [36] at Kaunas University of Technology and later expanded to include additional features. The latest variant of this feature set was devised here by combining feature subsets # 1–13 of [31] with MFCC and PLPCC features of [32]. For MFCC and PLPCC features the signal is pre-emphasized by 0.97 and frames are computed using the sliding 10 ms (440 samples) Hamming window with 5 ms overlap. The frame-based 19 MFCCs and 19 PLPCCs were characterized by 13 statistical functionals, resulting in a subset of 494 features. Combining 773 [31] and 494 [32] features formed the KTU feature set of 1267 features.

### jAudio features

The feature set # 16 was computed using the Java application jAudio [33] (version 0.4.5.1), which was developed as a standardized audio feature extraction system for automatic music classification. All features selected were frame-based with window size of 1024 (corresponding to ∼23.3 ms frame length) and window overlap of 50%. A detailed list of 1794 (138×13 local) features: 100 area (zeroth moment) estimates from 2D method of moments analysis of spectral data frames, 13 MFCC, 10 LPC, 4 spectral (centroid, flux, rolloff point, variability), 3 strongest frequency (via zero crossings, via spectral centroid, via FFT maximum), 2 partial-based spectral (centroid, flux), peak-based spectral smoothness, compactness, root mean square, fraction of low energy windows, relative difference function, and zero crossings.

### YAAFE features

The feature set # 17 was computed by yet another audio features extraction toolbox—YAAFE [34] (version 0.65). Default settings were left intact for the following list of 1885 (145×13 local) features: 24 loudness, 23 spectral crest factor per band, 23 spectral flatness per band, 13 MFCC, 12 shape statistics (4 envelope, 4 spectral, 4 temporal), 10 LSF, 10 OBSI, 9 OBSIR, 8 amplitude modulation, 6 spectral (decrease, flatness, flux, rolloff, slope, variation), 2 LPC, 2 perceptual (sharpness, spread), complex domain onset detection, energy, and zero-crossing rate.

### Tsanas features

The feature set # 18 contained various dysphonia measures and was dedicated initially specifically for PD screening. The Matlab code to compute these features is publicly available as Voice Analysis Toolbox (version 1.0) and the full list of 339 features is described in PhD thesis of [35]. Collection of audio features contains: jitter variants, shimmer variants, harmonic-to-noise ratio, noise-to-harmonics ratio, glottal quotient, glottal-to-noise excitation ratio, vocal fold excitation ratio, entropy of intrinsic mode functions from empirical mode decomposition, log energy, 13 MFCCs and their 1st and 2nd differences, de-trended fluctuation analysis, pitch period entropy and recurrence period density entropy.

## Methodology

Random forest (RF) [37] was used as a supervised algorithm to detect PD and also to fuse information in the form of soft decisions, obtained using various audio feature sets from separate modalities.

### Random forest

RF is a committee of decision trees, where the final decision is obtained by majority voting. The basic idea of RF is to combine many (*B* in total) unprunned CART (classification and regression tree) models, built on different bootstrap samples of the original dataset **X** and a random subset (of predetermined size *q*) of features *x*^1^, …, *x*^*p*^. For our experiments *B* was 5000 and after testing several specific values of *q* ($\sqrt[{}]{p}$, $2\cdot \sqrt[{}]{p}$, $\frac{1}{2}\cdot p$) the best performing (giving the lowest C_llr_) *q* was chosen.

RF is known to be robust against over-fitting and as the number of trees increases, the generalization error converges to a limit [37]. Low bias and low correlation are essential for the robust generalization performance of the ensemble. To get low bias, trees are unpruned (grown to the maximum depth). To achieve the low correlation of trees, randomization is applied.

RF is constructed in the following steps:

1. Choose the forest size *B* as a number of trees to grow and the subspace size *q* ≤ *p* as a number of features to provide for each tree node.
2. Draw a bootstrap sample (random sample with replacement) of the dataset, which generally results in $∼\frac{2}{3}\cdot n$ unique observations for training, thus leaving $∼\frac{1}{3}\cdot n$ for testing as the out-of-bag (OOB) dataset for that particular tree, where *n* is the number of observations in the dataset.
3. Grow an unpruned tree using the bootstrap sample. When growing a tree, at each node, *q* variables are randomly selected out of the *p* available.
4. Repeat steps 2 and 3, until the size of the forest reaches *B*.

The generalization performance of RF was evaluated using internal out-of-bag (OOB) validation, where each observation is classified only by the trees which did not have this observation in the bootstrap sample during construction. It is well known that the OOB validation provides an unbiased estimate of a test set error, similar to the leave-one-out scheme. Because of the “repeated measures” aspect, often arising in the phonation modality when each subject is represented by several recordings of voiced vowel, the sampling part of the Matlab implementation [38] had to be modified to ensure that all recordings of each subject are included either in a bootstrap sample or left aside as OOB. Added modification conforms to the leave-one-subject-out approach and helps to avoid biased evaluation when pathology detection intermingles with speaker detection. Additionally, the RF setting of stratified sampling was configured to preserve the class and gender balance of the full dataset in each bootstrap sample.

### Decision-level fusion

Individual RFs were built independently using various feature sets and OOB decisions of these individual experts were combined in a meta-learner fashion. RF was applied both as a base-learner and as a meta-learner. Therefore, outputs from the first stage base RFs are concatenated into a new feature vector, which becomes an input for the second stage meta RF. In the detection task, an input to the meta-learner is the difference between class posteriori probabilities computed by the base-learner. Given a trained base-learner, this difference is estimated as:

$$\begin{array}{c} d\left(\left\{t_{1},...,t_{b}\right\},x\right)=\frac{\sum_{i=1}^{b}f\left(t_{i},x,c=2\right)}{b}-\frac{\sum_{i=1}^{b}f\left(t_{i},x,c=1\right)}{b} \end{array}$$ (1)

where **x** is the object being classified, *b* is the number of trees *t*_1_, …, *t*_*b*_ in the RF for which observation **x** is OOB, *c* is a class label (1 corresponds to HC, 2 to PD), and *f*(*t_i_*, **x**, *c*) stands for the *c*-th class frequency in the leaf node, into which **x** falls in the *i*-th tree *t*_*i*_ of the forest:

$$\begin{array}{c} f\left(t_{i},x,c\right)=\frac{n(t_{i},x,c)}{\sum_{j=1}^{C}n\left(t_{i},x,c_{j}\right)} \end{array}$$ (2)

where *C* is the number of classes and *n*(*t_i_*, **x**, *c*) is the number of training data from class *c* falling into the same leaf node of *t*_*i*_ as **x**.

Additionally, for the purpose of visualization, a data proximity matrix Φ was obtained from the best meta-RF. Proximity matrix is constructed as follows: observations, represented by the meta-features, are run down each tree grown and the matrix element *ϕ*_*ij*_ is increased by one when two observations **x**_*i*_ and **x**_*j*_ are found in the same terminal node of the tree. After the meta-RF is constructed, proximities are obtained and divided by the total number of trees in the meta-RF. To project data into the 2D space, the proximity matrix Φ was converted through a simple 1 − Φ operation into a distance matrix and was provided as an input to the *t*-distributed stochastic neighbor embedding (*t*-SNE) algorithm [39] to implement dimensionality reduction. The main tunable parameter of *t*-SNE is perplexity, which controls the trade-off between concentrating on local versus global aspects of the data [40] and is comparable to the number of nearest neighbors in other manifold learning algorithms.

### Assessing detection

RF detector’s scores for OOB data were used to evaluate the goodness of detection. Votes of RF were converted to a proper score by dividing votes for a specific class from the total number of times the case was OOB, as in formula (1). Soft decision (score) instead of hard decision (predicted class) makes evaluation more precise by enabling visual summary of detection performance through the detection error trade-off (DET) curve, as recommended by [26]. A quick way to compare detectors with different DET curves is the equal error rate (EER)—the equilibrium point where curve intersects diagonal [41] and false positive rate (miss rate) becomes equal false negative rate (false alarm rate) or true positive rate (sensitivity) becomes equal true negative rate (specificity). The minimum cost of log-likelihood-ratio (C_llr_) is a comprehensive detection metric used here as the main criterion for model selection. The log-likelihood-ratio is the logarithm of the ratio between the likelihood that the target (PD person) produced the signal and the likelihood that a non-target (HC person) produced the signal. The DET curve, EER and C_llr_ measures were computed by the ROC convex hull method using the BOSARIS toolkit [42]. A well-calibrated and useful detector should provide C_llr_ < 1 and EER < 50%.

## Experimental results

The detection performance of individual feature sets was evaluated by estimating recording-based C_llr_ and EER measures. Then various unimodal and multimodal decision-level fusions were tested. Numbers corresponding to the minimum of each table column are denoted in ***bold italic*** font style.

### Individual feature sets

**Fig 1:**
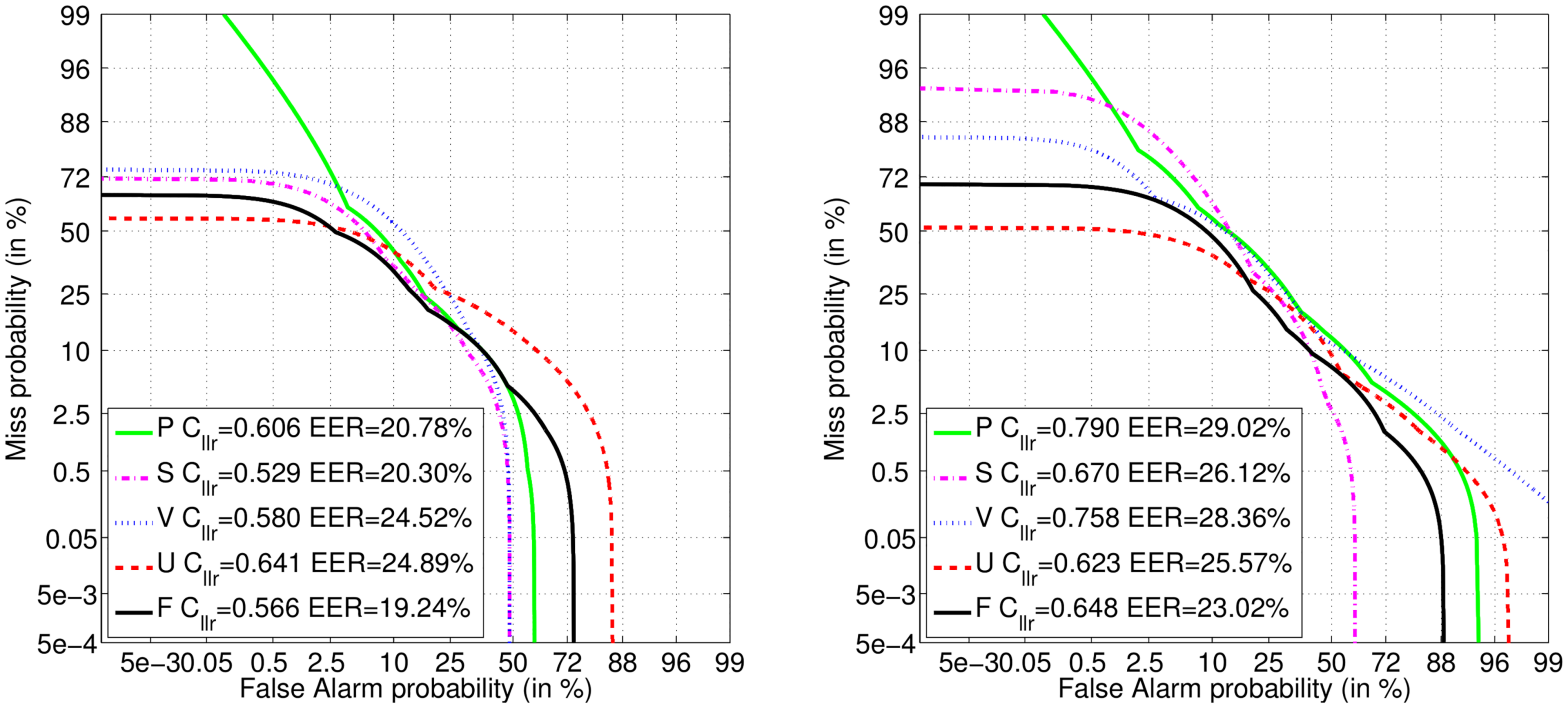
OOB detection performance by the DET curves. Microphone: AC (**left**) and SP (**right**). The best individual feature set (and corresponding modality): Essentia (P, S, V) and IS13_ComParE (U) using AC; Tsanas (P), jAudio (S), IS11_speaker_state (V) and YAAFE (U) using SP. Multimodal fusion (F) of all individual feature sets from all modalities.

Detection performance obtained using individual feature sets is summarized in Tables 4 and 5. DET curves of the best performing feature set (having the lowest EER) for each modality are provided in Fig 1. Essentia descriptors were the best using the AC channel for phonation, speech and voiced modalities, providing EER of 20.78%, 20.30%, and 24.52%, respectively. The best performance for unvoiced modality using the AC channel was EER of 24.89% obtained by IS13_ComParE features. The best individual performance using the SP channel for phonation, speech, voiced and voiced modalities was observed with Tsanas, jAudio, IS11_speaker_state and YAAFE features, providing EER of 29.02%, 26.12%, 28.36% and 25.57%, respectively. If instead of EER we consider C_llr_, the best feature set for the unvoiced modality of the AC channel is IS12_speaker_trait_compat and for the voiced modality of the SP channel is Tsanas. Phonation is often outperformed by the speech, especially in the SP channel, where the exception to this tendency is shown only by 2 feature sets (# 1–2) according to C_llr_ or 5 feature sets (# 1–4, 8) according to EER. Interestingly, the best individual performance in the AC channel was observed for the speech recording, but in the SP channel for the unvoiced part of the speech.

**Table 4:** OOB detection performance by C_llr_ using individual feature sets.

#	Feature set name	Modalities using AC channel				Modalities using SP channel			
		P	S	V	U	P	S	V	U
1	avec2011	0.802	0.693	0.782	0.708	0.791	0.806	0.871	0.683
2	avec2013	0.794	0.749	0.813	0.726	0.809	0.833	0.879	0.665
3	emo_large	0.744	0.758	0.768	0.724	0.834	0.775	0.856	0.760
4	emobase	0.718	0.737	0.758	0.666	0.857	0.776	0.833	0.637
5	emobase2010	0.837	0.854	0.735	0.777	0.830	0.778	0.792	0.734
6	IS09_emotion	0.906	0.778	0.807	0.734	0.842	0.804	0.839	0.742
7	IS10_paraling	0.841	0.833	0.750	0.777	0.832	0.738	0.787	0.723
8	IS10_paraling_compat	0.838	0.879	0.706	0.777	0.826	0.764	0.792	0.729
9	IS11_speaker_state	0.822	0.722	0.767	0.636	0.838	0.777	0.758	0.737
10	IS12_speaker_trait	0.822	0.724	0.735	0.649	0.822	0.741	0.773	0.766
11	IS12_speaker_trait_compat	0.814	0.727	0.758	***0.624***	0.816	0.739	0.795	0.734
12	IS13_ComParE	0.819	0.701	0.745	0.641	0.817	0.755	0.783	0.767
13	Essentia_descriptors	***0.606***	***0.529***	***0.580***	0.747	0.912	0.804	0.839	0.713
14	MPEG7_descriptors	0.665	0.623	0.798	0.753	0.910	0.844	0.911	0.745
15	KTU_features	0.810	0.770	0.780	0.767	0.930	0.805	0.837	0.707
16	jAudio_features	0.806	0.772	0.893	0.720	0.886	***0.670***	0.817	0.692
17	YAAFE_features	0.717	0.761	0.770	0.713	0.892	0.701	0.812	***0.623***
18	Tsanas	0.790	0.762	0.719	0.719	***0.790***	0.747	***0.749***	0.700

Notes. Microphone: AC—acustic cardioid, SP—smart phone. Modality: P—phonation, S—speech, V—voiced part of speech, U—unvoiced part of speech.

**Table 5:** OOB detection performance by EER (in %) using individual feature sets.

#	Feature set name	Modalities using AC channel				Modalities using SP channel			
		P	S	V	U	P	S	V	U
1	avec2011	30.65	27.40	31.38	28.46	30.74	34.08	36.67	26.96
2	avec2013	32.09	28.76	31.61	29.59	32.64	34.90	38.41	27.32
3	emo_large	25.74	29.58	28.57	30.64	29.17	30.18	34.33	32.00
4	emobase	24.14	26.41	27.82	25.54	32.59	32.78	36.15	26.07
5	emobase2010	31.84	35.76	32.22	35.71	30.89	30.17	31.34	31.34
6	IS09_emotion	37.03	28.06	33.86	32.56	34.26	32.17	33.82	28.33
7	IS10_paraling	32.59	34.01	31.71	35.20	31.89	30.53	32.62	31.54
8	IS10_paraling_compat	31.04	36.25	31.15	34.75	30.51	30.74	31.90	30.80
9	IS11_speaker_state	31.24	30.83	33.02	24.95	32.02	31.35	***28.36***	28.28
10	IS12_speaker_trait	32.74	30.14	31.90	26.87	31.93	30.35	31.91	31.08
11	IS12_speaker_trait_compat	30.51	29.66	31.43	25.51	31.51	29.85	31.65	31.34
12	IS13_ComParE	32.14	30.08	33.16	***24.89***	31.99	30.17	31.55	31.33
13	Essentia_descriptors	***20.78***	***20.30***	***24.52***	31.60	39.01	31.62	31.36	27.57
14	MPEG7_descriptors	21.25	22.19	32.26	31.38	38.54	32.36	37.25	27.11
15	KTU_features	29.22	30.11	29.53	31.64	43.11	29.10	33.22	29.13
16	jAudio_features	30.59	31.34	35.89	29.59	33.92	***26.12***	29.53	28.50
17	YAAFE_features	23.61	29.67	27.17	27.66	35.99	29.03	28.43	***25.57***
18	Tsanas	29.16	30.09	31.18	26.92	***29.02***	27.52	30.91	26.70

Notes. Microphone: AC—acustic cardioid, SP—smart phone. Modality: P—phonation, S—speech, V—voiced part of speech, U—unvoiced part of speech.

Variable importance analysis for the best performing feature sets is summarized in S1 File. Results for the AC phonation and speech modalities indicate the frequency band, Bark frequency band and the spectral statistics as the most important audio features. Results for the SP microphone indicate the MFCCs in speech and spectral statistics in unvoiced modality as the most important audio features.

### Decision-level fusion

Decision-level fusion of individual feature sets from all modalities helped to improve detection performance slightly according to EER (compare Table 5 with Table 6 and DET curves in Fig 1), where the best average EER was 19.27% for the AC and 23% for the SP channel. Meanwhile, according to C_llr_ no fusion variant could improve over performance of the best individual feature set from the single modality (compare 0.529 of AC speech and 0.623 of SP unvoiced in Table 4 with 0.553 of AC S+V+U and 0.646 of SP S+U in Table 6). Therefore, for the data investigated decision-level fusion remains of questionable effectiveness.

**Table 6:** Performance measures for 4 unimodal and 6 multimodal decision-level fusions.

Fusion	AC channel		SP channel	
	C_llr_	EER, %	C_llr_	EER, %
P	0.583 (0.004)	21.05 (0.16)	0.804 (0.004)	32.81 (0.26)
S	0.578 (0.006)	21.96 (0.22)	0.660 (0.007)	25.33 (0.28)
V	0.576 (0.004)	25.09 (0.50)	0.739 (0.005)	25.96 (0.25)
U	0.660 (0.007)	26.36 (0.55)	0.672 (0.004)	25.21 (0.42)
P+S	0.585 (0.004)	21.09 (0.22)	0.676 (0.006)	23.90 (0.38)
S+V	0.579 (0.004)	22.55 (0.24)	0.686 (0.005)	23.58 (0.38)
S+U	0.566 (0.006)	22.32 (0.26)	***0.646 (0.005)***	25.36 (0.35)
V+U	0.567 (0.005)	24.73 (0.34)	0.697 (0.007)	24.48 (0.65)
S+V+U	***0.553 (0.007)***	23.08 (0.39)	0.660 (0.007)	25.00 (0.49)
P+S+V+U	0.563 (0.004)	***19.27 (0.31)***	0.652 (0.006)	***23.00* (0.35)**

Notes. Fusion was repeated 99 times to estimate the mean (standard deviation). Microphone: AC—acustic cardioid, SP—smart phone. Modality: P—phonation, S—speech, V—voiced part of speech, U—unvoiced part of speech.

### Visualization by the 2D map

**Fig 2:**
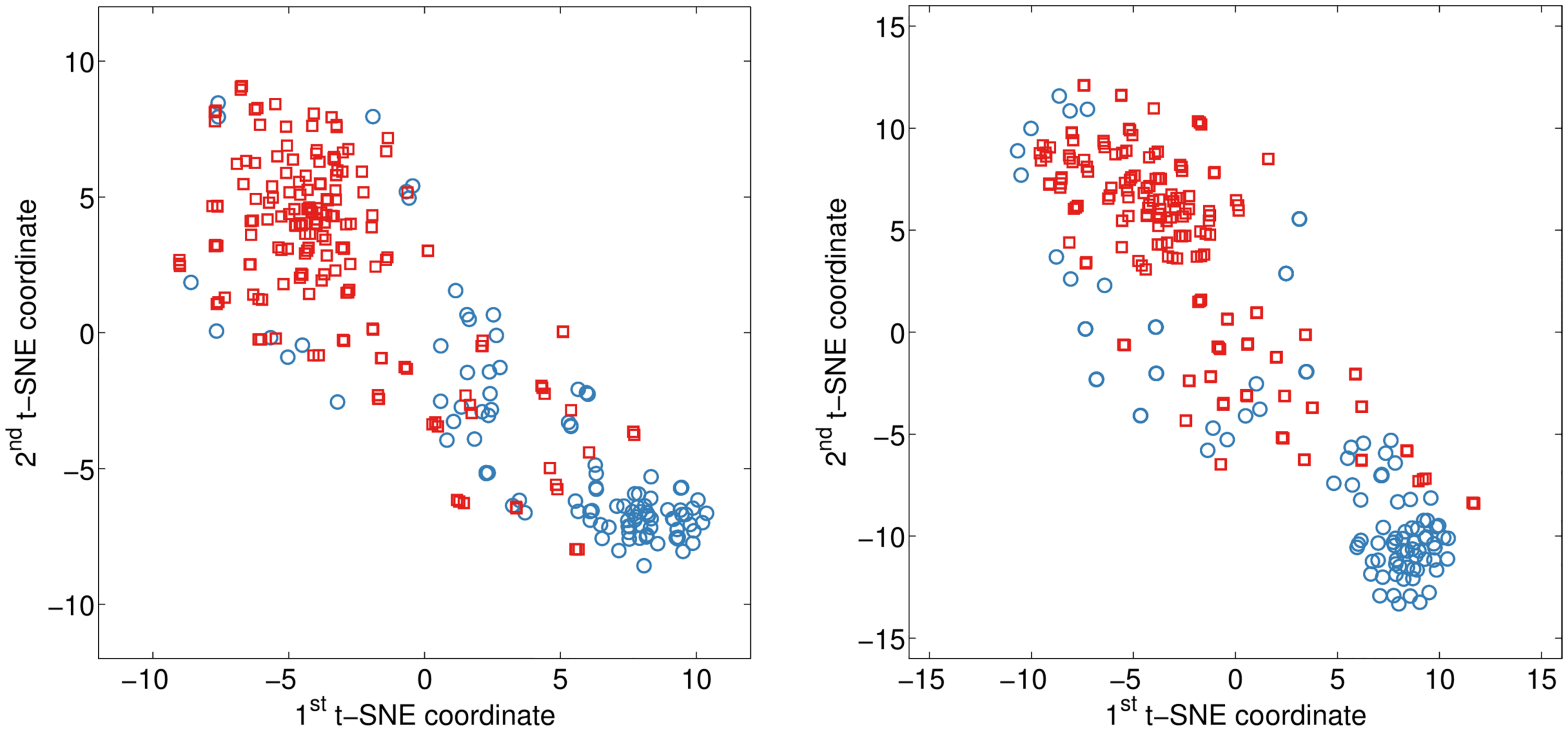
Visualization of the meta-RF proximity matrix by the *t*-SNE. Microphone: AC (**left**) and SP (**right**). Recording from: PD (designated by a red square □) and HC subject (designated by a blue circle ○).

The proximity matrix, obtained from the meta-RF, contains information about the pair-wise similarity between recordings with respect to the various feature sets. A compelling property of such a matrix is that only feature sets contributing to the construction of the meta-RF have an affect on similarity values. Therefore, the influence of unimportant, noisy feature sets gets reduced. Proximity matrices were obtained from the ultimate meta-RF model built fusing decisions from all individual feature sets and all modalities for the AC and SP channels. The perplexity parameter of the *t*-SNE algorithm was chosen empirically and set to 60. The representational error after 1000 iterations reached 0.313 for the AC and 0.305 for the SP channel. The resulting *t*-SNE visualizations are shown in Fig 2, where separate clusters of PD and HC subjects can be noticed.

Having a new recording with an unknown diagnosis, it could be converted to the audio features and fed to the first stage RFs, constructed on the individual feature sets. Then resulting decisions should be streamlined to the second stage meta-RF and its proximity matrix augmented with similarities of this unknown recording to the observations with available diagnosis. Running the *t*-SNE on a distance matrix, obtained from the augmented proximity matrix, would result in a new recording located as a distinct point in the 2D space among the known cases (among the points in Fig 2). Exploring location of the new recording with respect to the points in the vicinity can be a useful data-driven exploratory approach for PD screening.

## Conclusions and future directions

The best individual feature set was Essentia when using speech modality of the AC microphone and YAAFE when using unvoiced modality of the SP microphone, achieving EER of 20.30% and 25.57%, respectively. Speech signal tends to outperform phonation in the PD detection task when using the SP microphone. Splitting of speech signal into voiced / unvoiced modalities, as recommended by [17], was found to be useful in the SP case.

Fusion of all feature sets and modalities resulted in EER of 19.27% for the AC microphone and EER of 23% for the SP microphone. Improvement from fusion was evident only according to EER, but according to the more comprehensive C_llr_ measure fusion is not effective for the data analysed. The non-linear mapping of proximity matrix obtained from the meta-RF into the 2D space was shown to enrich medical decision support by allowing to spot similar cases conveniently.

Detection performance was consistently better for the AC than for the SP microphone. Nonetheless, text-dependent speech recordings of SP quality and especially their unvoiced part have potential for PD detection. More vocal exercises, like rapid speech movements through succession by the diadochokinetic task of /*pa*/-/*ta*/-/*ka*/ repetition, could also be useful. Additional information is worth considering by tracking an accelerometer signal in a posture test [19] of standing still and/or holding device in a hand with an arm extended or in a gait test [19] of walking. Tapping and reaction time tests [19] or drawing of an Archimedean spiral [43] are an interesting type of tactile tasks which could be recorded using a hand-held device. Fusion of information from diverse non-invasive modalities could help to develop an efficient SP-based tool for PD screening.

## Ethical statement

The study protocol has been approved by Kaunas Regional Bioethics Committee (P2-24/2013). Written informed consent was obtained from the study participants, patient identifiers were removed to ensure anonymity.

## Supporting information

S1 File Variable importance analysis in the task of PD detection is reported for the best performing RF and meta-RF models. (PDF)

S2 File Experimental data, in the form of extracted audio features from voice and speech recordings obtained through acoustic cardioid microphone channel. (ZIP)

S3 File Experimental data, in the form of extracted audio features from voice and speech recordings obtained through internal smart-phone microphone channel. (ZIP)
