# Supplementary material for: Detecting Parkinson’s disease from sustained phonation and speech signals
Source: PLoS One. 2017 Oct 5;12(10):e0185613. doi: 10.1371/journal.pone.0185613 (PMC5628839; doi:10.1371/journal.pone.0185613)
Supplement: S1 File — (PDF) [file pone.0185613.s001.pdf]

# S1 Appendix: Variable importance analysis

Features were investigated by performing the permutation-based variable importance analysis using the mean decrease in accuracy as the variable importance measure. Values of each feature are permuted several times and the mean difference in the RF performance on OOB data is reported. In case of important variable, permutation should break ties (intrinsic relationship between variable values and class labels) to the point where accuracy decreases noticeably. Even though accuracy-based importance is less biased than Gini-based one towards predictors with a large number of unique values [1], it still has some limitations. Original accuracy-based permutation importance is used here, but we forewarn of its tendency to overestimate importance of correlated predictors [2].

Due to the presence of multitude audio features, in case of individual RF, and meta-features from several modalities, in case of meta-RF, features (or meta-features) were grouped into meaningful groups. Estimated mean decrease in accuracy was summed for each group of audio features to provide a bar chart or coloured and stacked for each type of meta-features.

Essentia descriptors from phonation and speech modalities were investigated for the AC channel (see Fig S1-1). jAudio features from speech modality and YAAFE features from unvoiced modality were investigated for the SP channel (see Fig S1-2). For meta-RF, fusion of all modalities (P+S+V+U) was considered to explore which feature sets were the most effective in decision-level fusion (see Fig S1-3).

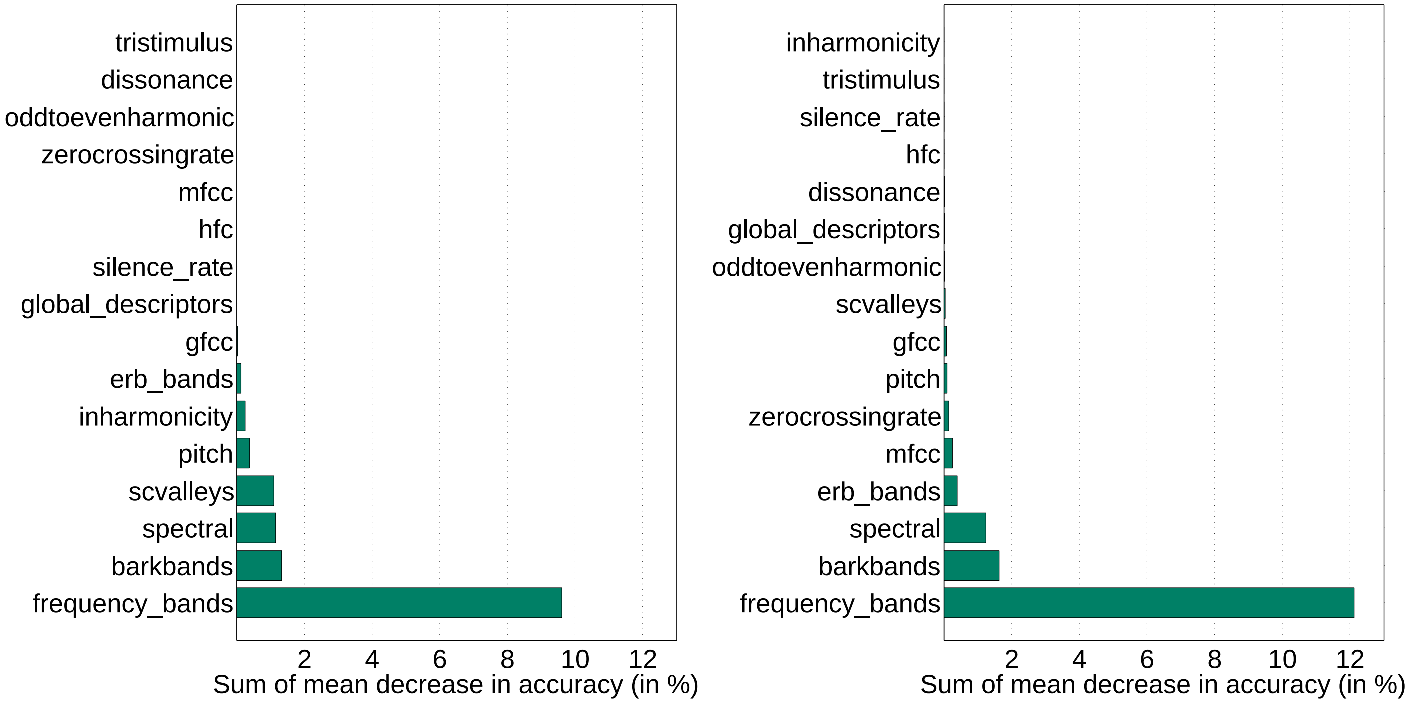


**Fig S1-1. RF permutation-based variable importance for the AC microphone.** Summarized are importances of Essentia descriptors from phonation (**left**) and speech modality (**right**).

Results of Essentia descriptors for the AC microphone in Fig S1-1 show that frequency and Bark frequency statistics together with spectral measures were the most important for PD detection in both phonation and speech modalities. Spectral contrast valleys have notably higher importance in phonation than in speech modality. Results of

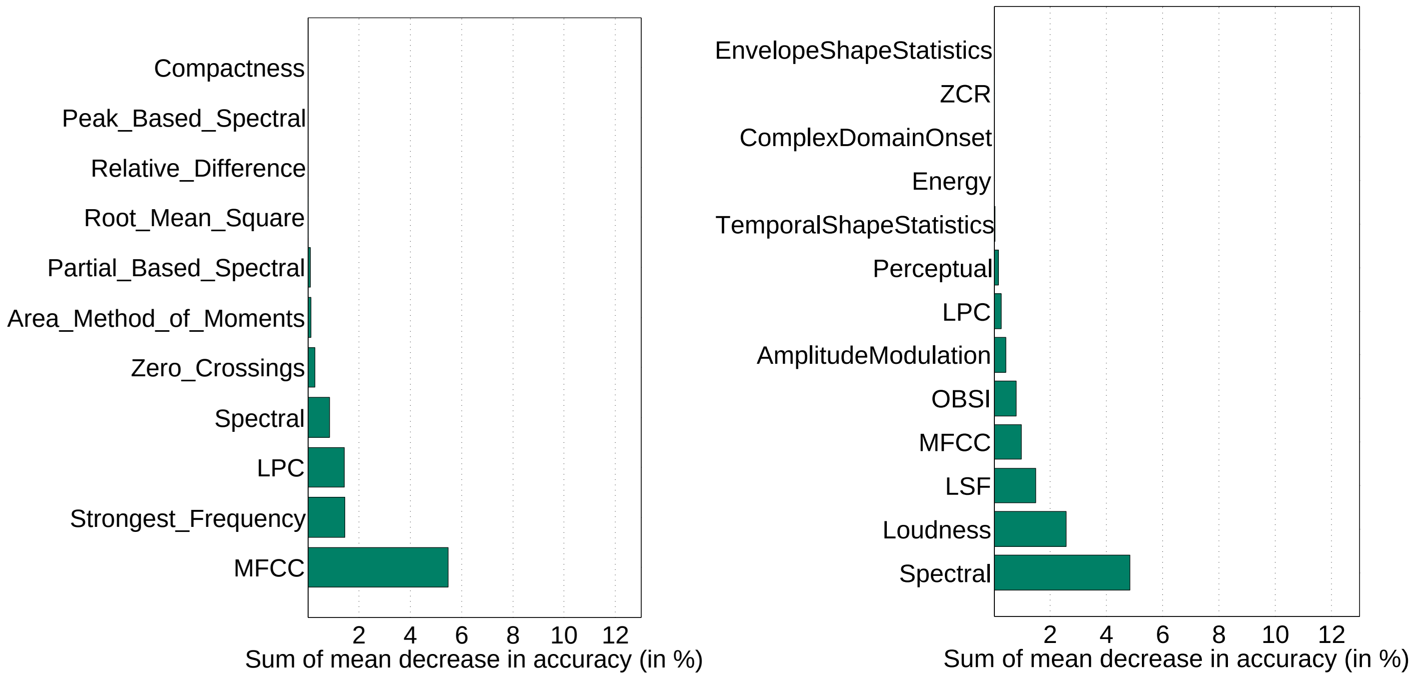


**Fig S1-2. RF permutation-based variable importance for the SP microphone.** Summarized are importances of jAudio features from speech (**left**) and YAAFE features from unvoiced modality (**right**).

jAudio and YAAFE features for the SP microphone in Fig S1-2 show highest importance of MFCCs, LPCs and strongest frequency statistics in speech, but spectral, loudness and line spectral frequency (LSF) measures in unvoiced modality.

Variable importance of meta-RF, shown in Fig S1-3, to some extent reflect rankings one could derive from Table 4. For AC microphone, Essentia descriptors are the most informative feature set in all modalities, except unvoiced, followed by MPEG7_descriptors. In AC unvoiced modality, the most informative is OpenSMILE IS12_speaker_trait_compat feature set. For SP microphone, top-ranked feature sets are jAudio, YAAFE and Tsanas, followed by avec2013 (having the largest importance in unvoiced modality). It is rather surprising that for the AC channel Essentia descriptors are the most important, but the very same feature set for the SP channel performs the worst. Also, for the SP case, where improvement in EER by using fusion was more noticeable, speech and unvoiced modalities dominate importance scores and more feature sets reach 2% importance, if compared to the AC case.

# References

1. Strobl C, Boulesteix AL, Zeileis A, Hothorn T. Bias in random forest variable importance measures: Illustrations, sources and a solution. BMC Bioinformatics. 2007;8(1):25. doi:10.1186/1471-2105-8-25.

2. Strobl C, Boulesteix AL, Kneib T, Augustin T, Zeileis A. Conditional variable importance for random forests. BMC Bioinformatics. 2008;9(1):307. doi:10.1186/1471-2105-9-307.

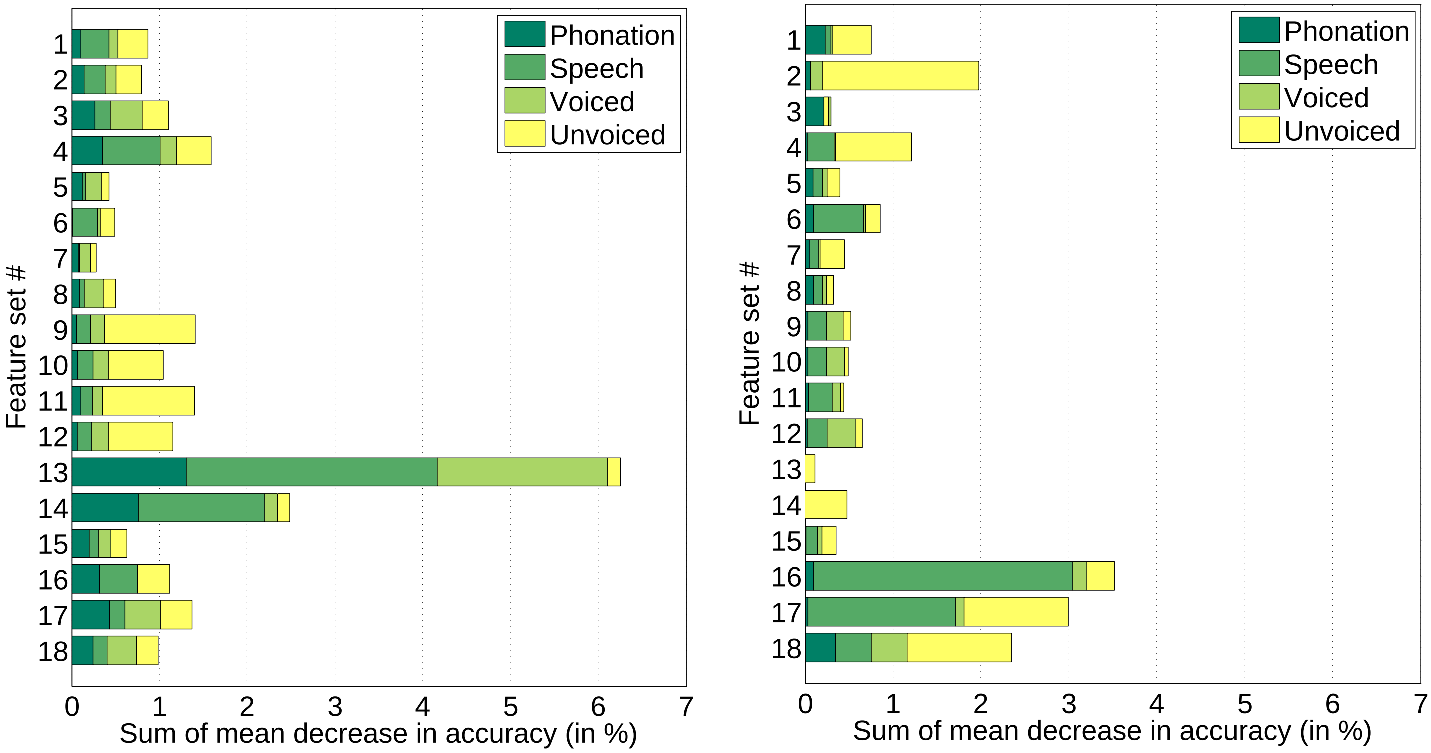


**Fig S1-3. Meta-RF permutation-based variable importance.** Microphone: AC (**left**) and SP (**right**). Contribution of investigated modalities in a stacked bar is coded by different colors.
